# Prevalence, Infectious Characteristics and Genetic Diversity of *Staphylococcus aureus* and Methicillin-Resistant *Staphylococcus aureus* (MRSA) in Two Raw-Meat Processing Establishments in Northern Greece

**DOI:** 10.3390/pathogens11111370

**Published:** 2022-11-17

**Authors:** Dimitrios Komodromos, Charalampos Kotzamanidis, Virginia Giantzi, Styliani Pappa, Anna Papa, Antonios Zdragas, Apostolos Angelidis, Daniel Sergelidis

**Affiliations:** 1 Laboratory of Food Hygiene-Veterinary Public Health, School of Veterinary Medicine, Aristotle University of Thessaloniki, 54124 Thessaloniki, Greece; 2 Hellenic Agricultural Organization—DIMITRA, Veterinary Research Institute of Thessaloniki, 57001 Thermi, Greece; 3 Department of Microbiology, Medical School, Aristotle University of Thessaloniki, 54124 Thessaloniki, Greece; 4 Laboratory of Safety and Quality of Milk and Dairy Products, School of Veterinary Medicine, Aristotle University of Thessaloniki, 54124 Thessaloniki, Greece

**Keywords:** *Staphylococcus aureus*, MRSA, meat products, antimicrobial resistance, virulence genes, biofilms, Pulsed Field Gel Electrophoresis (PFGE), *spa* typing, next-generation sequencing

## Abstract

In the present study, we investigated the isolation frequency, the genetic diversity, and the infectious characteristics of *Staphylococcus aureus* and methicillin-resistant *S. aureus* (MRSA) from the incoming meat and the meat products, the environment, and the workers’ nasal cavities, in two meat-processing establishments in northern Greece. The isolated *S. aureus* strains were examined for their resistance to antimicrobials, carriage of the *mec*A and *mec*C genes, carriage of genes encoding for the production of nine staphylococcal enterotoxins, carriage of the Panton–Valentine Leukocidin and Toxic Shock Syndrome genes, and the ability to form biofilm. The genetic diversity of the isolates was evaluated using Pulsed Field Gel Electrophoresis (PFGE) and *spa* typing. *S. aureus* was isolated from 13.8% of the 160 samples examined, while only one sample (0.6%) was contaminated by MRSA carrying the *mec*A gene. The evaluation of the antimicrobial susceptibility of the isolates revealed low antimicrobial resistance. The higher resistance frequencies were observed for penicillin (68.2%), amoxicillin/clavulanic acid (36.4%) and tetracycline (18.2%), while 31.8% of the isolates were sensitive to all antimicrobials examined. Multidrug resistance was observed in two isolates. None of the isolates carried the *mec*C or *luk*F-PV genes, and two isolates (9.1%) harbored the *tst* gene. Eight isolates (36.4%) carried the *seb* gene, one carried the *sed* gene, two (9.1%) carried both the *sed* and *sei* genes, and one isolate (4.5%) carried the *seb, sed* and *sei* genes. Twenty-one (95.5%) of the isolates showed moderate biofilm production ability, while only one (4.5%) was characterized as a strong biofilm producer. Genotyping of the isolates by PFGE indicates that *S. aureus* from different meat-processing establishments represent separate genetic populations. Ten different *spa* types were identified, while no common *spa* type isolates were detected within the two plants. Overall, our findings emphasize the need for the strict application of good hygienic practices at the plant level to control the spread of *S. aureus* and MRSA to the community through the end products.

## 1. Introduction

*Staphylococcus aureus* is part of the skin and mucous microbiota of humans, with the nasal cavity constituting the most common carriage site. The nasal cavity is regarded as the source of colonization of secondary body sites, such as the hands [1]. *S. aureus* is also an important versatile pathogen causing various types of infections, ranging from skin and soft-tissue infections to life-threatening septicemia and toxin-mediated diseases, such as staphylococcal food poisoning (SFP), toxic shock syndrome toxin 1 (TSST-1) and staphylococcal scalded skin syndrome (SSSS) [2]. Moreover, methicillin-resistant *S. aureus* (MRSA) is of particular importance for both public and animal health. There are three groups among MRSA strains: hospital-associated MRSA (HA-MRSA), community-associated MRSA (CA-MRSA) and livestock-associated MRSA (LA-MRSA) [3,4]. Transmission of LA-MRSA occurs mainly in humans with occupational exposure to livestock [4]. It is also likely that LA-MRSA may be transmitted through contaminated meat products [3,5].

The presence of *S. aureus* and MRSA in the environment of livestock farms and slaughterhouses and the risk of their transmission to the meat and the workers in these settings has been of great concern to the scientific community [6,7,8,9,10,11].

Pathogenic bacterial attachment and the formation of persistent biofilms in food-processing establishments represent an important risk for food contamination and the spread of pathogens in the community [12,13]. *S. aureus* can develop persistent biofilms on both biotic and abiotic surfaces [14]. The co-existence of different species within the biofilm matrix of staphylococci, and especially of *S. aureus*, with numerous benefits for them, such higher antimicrobial tolerance, has been well documented [15,16]. Preventing the development of biofilms on food-processing surfaces is critical for food safety.

Given their ever-changing epidemiology of *S. aureus* and MRSA, updated information is needed for the implementation of effective control measures [17,18]. The identification of possible sources and routes of meat contamination in the processing facilities contributes significantly to the evaluation and improvement of preventive measures targeting their transmission to the final products and therefore their dispersion in the community. In this study, to obtain more insights into the epidemiology of *S. aureus*, in meat-processing plants, we investigated the prevalence of *S. aureus* and MRSA in the incoming meat and meat products, the environment, and the workers’ nasal cavities of two meat-processing establishments in northern Greece. All *S. aureus* isolates were tested for their resistance to certain antimicrobials. In addition, staphylococcal strains were tested by molecular methods for detection of the *mec*A, *mec*C, *pvl*, *tst-1* and enterotoxin genes, while their ability to form biofilms was also investigated. Their genetic relatedness was evaluated using Pulsed Field Gel Electrophoresis (PFGE) and *spa* typing.

## 2. Results

### 2.1. Isolation of S. aureus

*S. aureus* was isolated from 22 of the 160 (13.8%) samples examined from the two meat-processing establishments. The isolation frequency was 23.3% (14/60) and 8.0% (8/100) for plant F and Z, respectively (Table 1). The isolation frequency of *S. aureus* in descending order was 37.5% (6/16) from ground meat products, 25% (5/20) from workers, 12.3% (7/57) from equipment, 10.0% (2/20) from incoming pork meat, 8.3% (1/12) from incoming bovine meat, and 4.5% (1/22) from infrastructure. The isolation frequency of *S. aureus* was 8.1% (3/37) in samples from the incoming meat (bovine, porcine and ovine), and 25.0% (6/24) in samples from the processed raw meat products (ground meat and non-ground meat products), respectively (Table 1).

### 2.2. Antimicrobial Resistance of S. aureus

**Figure 1:**
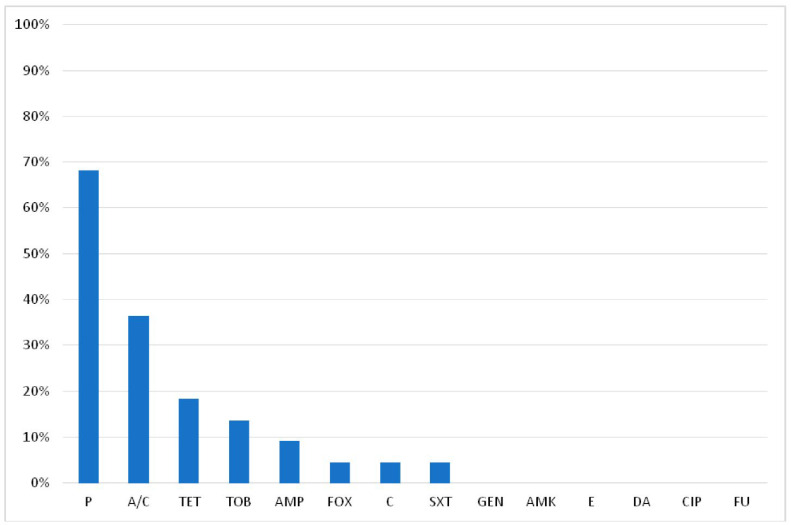
Frequency of antimicrobial resistance of *S. aureus* isolates from two meat-processing plants.

The frequency of resistance (in descending order) of the *S. aureus* strains to the tested antimicrobials was: penicillin (P) (68.2%), amoxicillin/clavulanic acid (A/C) (36.4%), tetracycline (TET) (18.2%), tobramycin (TOB) (13.6%), ampicillin (AMP) (9.1%), cefoxitin (FOX) (4.5%), chloramphenicol (C) (4.5%) and sulfamethoxazole/trimethoprim (SXT) (4.5%) (Figure 1). One isolate (Z112) being resistant to cefoxitin was phenotypically considered as MRSA; seven isolates (31.8%) were susceptible to all antimicrobials examined while 45.4% (10/22) of the *S. aureus* was resistant to more than one antimicrobial (Table 2). In total, seven antimicrobial resistance profiles (ARPs) were found, with the profiles ‘P’ and ‘P, A/C’ being the most frequent (22.7% each). Two isolates (F40 and Z112) were identified as multidrug resistant (MDR), exhibiting resistance to three and six different classes of antimicrobials, respectively.

### 2.3. Carriage of the mecA, mecC, tst, lukF-PV and Enterotoxin Genes

The *mec*C and *luk*F-PV genes were not detected in any *S. aureus* isolate. Two isolates carried the *tst* gene: one from equipment of plant F and the other from a worker’s nasal cavity from plant Z (Table 2). Ten of the isolates (45.6%) did not carry any of the examined staphylococcal enterotoxin genes, eight of the isolates (36.4%) carried only the *seb* gene, two (9.1%) carried the *sed* and *sei* genes and one (4.5%) carried the *seb, sed* and *sei* genes (Table 2). Of the 12 isolates that carried enterotoxin genes, five (41.7%) were isolated from equipment surfaces, four (33.3%) from workers’ nasal cavities and three (16.7%) from meat products. The MRSA isolate (Z112) that originated from a sample of meat product in plant Z carried the *mec*A gene, but it did not harbor any of the remaining virulence genes examined (Table 2).

### 2.4. Biofilm Formation Ability

All except one of the *S. aureus* isolates (21/22, 95.5%) were classified as moderate biofilm producers and the remaining isolate was classified as a strong biofilm producer (Table 2).

### 2.5. Spa Typing

The *spa* typing of the 22 *S. aureus* strains revealed ten *spa* types, one of which was detected for the first time. About half the strains (9/22) belonged to *spa* type t084, two strains belonged to type t091 and four strains belonged to the new *spa* type. The sequence of the novel *spa* type (repeats 07-23-12-12-21-17-13-34-33-34) was confirmed in the whole genome sequence; actually, it is similar to the spa type t2414 (07-23-12-12-21-17-34-33-34) with one additional repeat (repeat 13). The other seven isolates belonged to t012, t197, t499, t774, t891, t899 and t1510 (Table 2). The MRSA strain belonged to *spa* type t899 and was not typeable by PFGE. This *spa* type belongs either to CC398 or to CC9 [19]. The five *S. aureus* isolates from the nasal cavities of workers were assigned to five different spa types (t084, t197, t891, t1510, ‘new’).

### 2.6. Pulsed Field Gel Electrophoresis Typing

**Figure 2:**
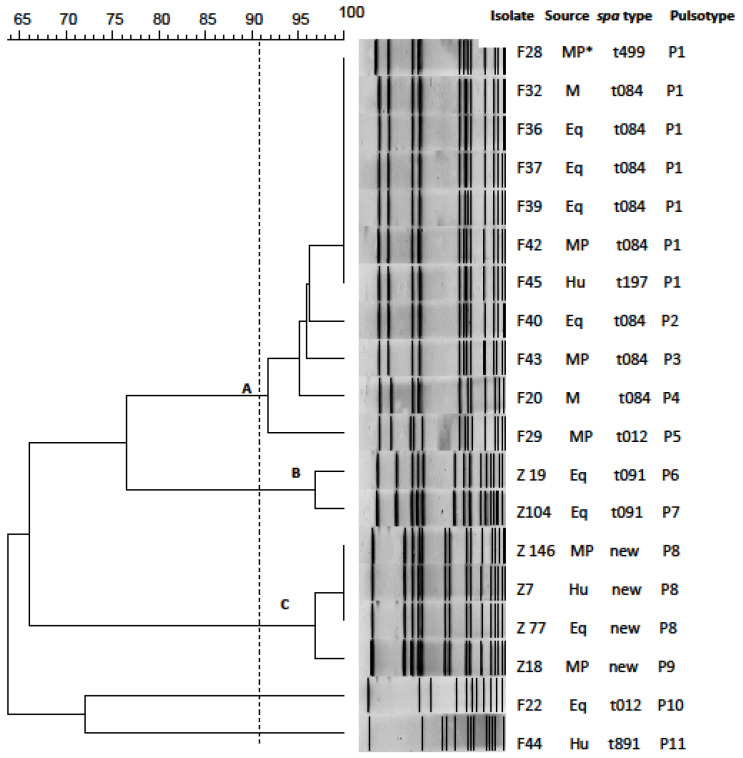
PFGE dendrogram of the *S. aureus* isolates. * MP = meat product, M = incoming meat, Eq = equipment, Hu = human.

Nineteen of the staphylococcal isolates were typeable by PFGE revealing 11 distinct pulsotypes (Figure 2). At a similarity level of 91%, most of the *S. aureus* isolates (57.9%, 11/19) were grouped into the cluster A; isolates from the same processing plant were assigned into separate clusters (A, B, and C). Two isolates from plant F (F22 and F44) were not assigned to any of the three clusters (Figure 2). Within clusters, common pulsotypes among isolates originating from human (Hu), meat (M), meat product (MP), and equipment (Eq) samples were identified: within cluster A, pulsotype P1 was shared by Hu, M, MP and Eq isolates, while within cluster C, pulsotype P8 was shared by Hu, MP and Eq isolates.

### 2.7. Next-Generation Sequencing

Analysis of the whole genome sequence of the two isolates (Z77 and Z112) showed that Z77, which was sensitive to all antibiotics tested, did not carry any antimicrobial resistance gene. Regarding the isolate Ζ112, which was resistant to several antibiotics, genes conferring resistance to beta-lactams (*mec*A, *bla*Z), chloramphenicol (*fex*A), tetracycline (*tet*M) and trimethoprim (*dfr*C) were detected; however, no gene associated with resistance to tobramycin was detected (Table 3).

*Spa* typing confirmed the type t899 in the Z112 isolate, while it was not possible to be defined in the Z77 isolate. The Z112 isolate belonged to sequence type ST398 and the Z77 isolate to ST97, respectively. Among the toxin genes we found, *hlg*A, *hlg*B, and *hlg*C were encoding for gamma-hemolysin components A, B and C, respectively, while *luk*D and *luk*E encode for leucotoxins D and E; the exoenzyme genes *aur*, *spl*A, *spl*B, and *spl*E encode for the aureolysin and serine protease-like proteins A, B and E, respectively; finally, *sak* and *scn* genes encode for the host immune defense proteins staphylokinase and staphylococcal complement inhibitor.

## 3. Discussion

### 3.1. Isolation, Antimicrobial Susceptibly and Biofilm Formation Ability of S. aureus and MRSA Isolates

Considering that bibliographic data on the prevalence of staphylococci in the products and in the environment of meat-processing establishments are limited, the main objective of this study was to report the prevalence of *S. aureus* and MRSA in two meat-processing establishments in northern Greece. In general, compared to meat-processing establishments, a higher detection frequency of *S. aureus* and MRSA has been reported for beef, pork and poultry meat products at the retail level [20]. In the present study, *S. aureus* was isolated from 13.8% of the samples examined, while only one sample of ground meat (0.6%) was contaminated with MRSA. These detection frequencies are lower than the estimates of 29.2% and 3.2% for *S. aureus* and MRSA, respectively, which were reported in a global meta-analysis of different meat products [20]. In samples taken from equipment surfaces of the meat-processing plants, the overall *S. aureus* isolation frequency was 12.3%, being comparable with a previous Spanish study, which reported a prevalence of 15.5% on equipment surfaces of a pork-processing plant [21]. However, further data concerning the *S*. *aureus* isolation frequency from equipment surfaces are scarce in the literature. In the personnel of the two meat-processing establishments, the isolation frequency of *S. aureus* was 25%. This finding is not surprising, since the detection of *S. aureus* in the population is around 20% and 60% in persistent and occasional nasal carriers, respectively [22]. Personnel in slaughterhouses and meat-processing plants are among the groups of workers with the highest incidence of occupational injuries. Injuries that lead to the disruption of skin-tissue integrity [23,24] as well as the frequent exposure to materials of animal origin (nasal secretions, gastrointestinal contents, etc.) favor the contamination of workers with *S. aureus* [11,25]. On the other hand, none of the samples from workers tested positive for MRSA. Drougka et al. [8] reported the isolation of MRSA from 4.2% of slaughterhouse workers in Greece, where the prevalence in the general population is low (0.94%) [26]. In our study, the lack of detection of MRSA in the samples obtained from workers at the examined facilities is likely due to the small number of samples examined. However, even the mere presence of *S. aureus* and MRSA on the surface of meat constitutes a potential risk for their transmission to humans, particularly to those who work in meat-processing companies [6].

To gain insight into the identification of specific phenotypic and genomic characteristics of *S. aureus* isolated from the meat-processing plants, we investigated their antimicrobial susceptibility, biofilm formation capacity and carriage of enterotoxin genes. The highest resistance frequency was observed to P (68.2%), A/C (36.4%), TET (18.2%) and TOB (13.6%). Seven distinct antimicrobial resistance profiles were revealed, with the profiles ‘P’ (22.7%) and ‘P, A/C’ (22.7%) being the most frequent. Finally, two MDR isolates (F40 and Z112) were revealed. The presence of antimicrobial-resistant strains of *S. aureus* and MRSA has been reported in various foods as well as in meat [8,27] and meat products [28,29] and multiple profiles of antimicrobial resistance are often observed among the *S. aureus* isolates [30]. The high resistance frequencies of *S. aureus* and MRSA isolates from livestock, meat and food handlers to P and TET, ranging from 60.9% to 100%, and from 5.6% to 89.1% respectively, have been reported by numerous studies worldwide (USA [29], Lithuania [31], Greece [8,32], Italy [33], Nigeria [34], China [35] and Korea [36]). The frequent resistance to penicillins and tetracyclines is apparently related to the widespread use of these antimicrobials due to their low cost, ease of access (without a prescription) and administration. We also observed a high (80%) resistance frequency to P among the *S. aureus* isolates originating from the workers’ nasal cavities (in four out of five positive workers) (Table 2). These findings are similar to those previously reported (70% [37], 48% [38], 57.9% [39]). On the other hand, the sensitivity of the workers’ *S. aureus* isolates to TET in the present study may be due to the infrequent use of tetracyclines in human clinical practice [40].

Previous studies have shown the presence (2.9%) of MDR MRSA isolates in turkey and pork meat [41]. Interestingly, the single MRSA isolate in our study (from ground pork meat) was characterized as MDR, showing resistance to six antimicrobial classes. Overall, the evaluation of antimicrobial susceptibility revealed a high frequency of resistance to specific antimicrobials as well as variable ARPs among the *S. aureus* isolates. Therefore, our findings emphasize the need for preventive measures to control the dispersion of antimicrobial resistance along the entire food production chain [42] with strict application of proper hygiene and industrial practices.

All *S. aureus* isolates were characterized by moderate biofilm-forming capabilities in polystyrene microtiter plates. In line with our findings, previous studies have reported the ability of biofilm formation by *S. aureus* isolates from raw meat [43], from several food-processing facilities [44,45] and from food of animal origin [35]. The ability of *S. aureus* to produce biofilms and their coexistence with saprophytic microorganisms within these biofilms result in persistent contamination sources in food-processing facilities [16,46].

### 3.2. Enterotoxin Gene Carriage of S. aureus Isolates

In the present study, 54.5% of the *S. aureus* isolates carried at least the *seb* or *sed* genes alone, together or in combination with *sei* (Table 2). Similarly high frequencies of enterotoxin gene carriage (51.3%) were reported for isolates from food of both animal and plant origin in China (*sec* (38.5%), *seg* (19.7%), *sej* (16.2%), *see* (12.8%), *sea* (11.1%), *seb* (10.3%), *sei* (4.3%), *sed* (3.4%) and *seh* (1.7%)) [47]. Normanno et al. [48] reported that 45.2% of the *S. aureus* isolates from meat and meat products at the retail level in Italy could produce at least the ‘classic’ enterotoxins (SEA (30.3%), SEB (7.6%), SEC (51.5%), SED (6.1%), SEA+SEB (1.5%), SEA+SED (3.0%)). In the same study, 50% of the isolates from surfaces in contact with meat could also produce SEC.

Four of the five (80%) *S. aureus* strains isolated from the workers’ nasal cavities were enterotoxigenic and five of the seven (71%) were from the equipment surfaces. In a study conducted in Hong Kong, 40% of the *S. aureus* isolates from the nasal cavities of meat handlers in mass-catering kitchens carried at least one enterotoxin gene, with *sea* (20%) and *seb* (11%) being the most frequently detected [49]. Workers carrying enterotoxigenic strains of *S. aureus* are recognized as the main source of their spread in food either through direct contact or indirectly through respiratory secretions [50].

The application of good hygiene practices during food processing and the effort to control and limit the population of staphylococci to low levels in foods are the most important measures for the prevention of staphylococcal intoxications [51].

### 3.3. Carriage of Genes Encoding for the Toxic Shock Syndrome and Panton–Valentine Leukocidin Toxins

The *tst* gene was detected in a *S. aureus* strain isolated from a worker’s nasal cavity and in one sample obtained from processing equipment. This low-isolation frequency (9.1%) is in line with the results of other studies. A zero-detection frequency was reported for isolates recovered from workers of a slaughterhouse in Greece and food handlers in Lebanon, respectively [8,52]. A low prevalence (5.3%) was also reported in samples taken from the hands of workers in restaurants in Spain [53]. These *tst* gene detection frequencies in workers are considerably lower compared to those reported for the general population in several countries, ranging from 15.8% to 40.0% (Brazil 15.8% [54], Madagascar 21.0% [55], Iran 22.8% [56], Spain 28.3% [57] and Egypt 40.0% [58]). There are limited reports on *tst* gene carriage among *S. aureus* isolates from processing surfaces. Sahin et al. [59] reported one *S. aureus* strain (isolated from surface samples in food-processing establishments in Turkey) carrying the *tst* gene, while Sospedra et al. [53] did not recover any *S. aureus* isolates carrying the *tst* gene from surfaces and equipment in restaurants in Spain.

In the present study, none of the *S. aureus* isolates carried the *luk*F-PV gene. Weese et al. [60] reported that none of the MRSA isolates from raw meat and its preparations in Canada carried this gene. On the contrary, Gutierrez et al. [45] reported that 10.1% of the *S. aureus* isolates from ground beef and pork chops in Colombia carried the *luk*F-PVL gene.

Given (a) the limited pertinent available literature internationally, (b) the fact that food handlers and processing surfaces are important sources of food contamination, and (c) the severity of clinical manifestations associated with TSST-1 and PVL intoxications, the low detection frequency of *S. aureus* isolates carrying these genes does not justify complacency.

### 3.4. Genetic Diversity of S. aureus Isolates

Another scope of this study was to reveal the genetic diversity of the *S. aureus* isolates to better understand the transmission routes and infection sources of this pathogen in meat-processing establishments. Ten different *spa* types were identified based on *spa*-typing; among them, five *spa* types (t197, t499, t774, t899 and t1510) were detected for the first time in Greece. Most of the strains belonged to the *spa* type t084 (40.9%), four (18.2%) to a new *spa* type and two (9.1%) to t091 (Figure 2). According to the Ridom Spa Server, the global spread of *spa* types t084 and t091 is quite high (1.74% and 0.99%, respectively). The most prevalent *spa* type (t084) was detected in all types of samples from plant F (Figure 2). Methicillin-sensitive *S. aureus* (MSSA) strains of this type have been isolated from several sources globally [57,61,62]. The *spa* type t091 represents the basic type of the clonal complex CC7 and has been detected as either MSSA or MRSA from various sources [57,63,64,65,66]. In addition, the *spa* type t012, which was identified in one isolate in our study, displays high global spread (1.53%). The *spa* types t197, t499, t774, t891, t899 and t1510 show a relatively low global spread, ranging between 0.02% and 0.31%. Interestingly, *S. aureus* isolates from workers belonged to different *spa* types (t084, t197, t891, t1510 and to a new type), which are usually isolated from human clinical samples [67,68,69]. The only MRSA isolate in the present study belonged to *spa* type t899. To our knowledge, and according to the data available in the Ridom Spa Server, this *spa* type is detected for the first time in Greece, and its global spread is 0.31%, mainly in Italy and other European countries. Originally, this *spa* type was related with MLST type ST9, but recently, it is most often related with ST398, which is the predominant LA-MRSA lineage in Europe [19,70]. Although t011 and t034 still constitute the main *spa* types associated to CC398, isolates belonging to *spa* type t899 have been reported as the cause of sporadic illness in humans, and they are increasingly being described in the human and veterinary medical literature [71,72,73,74,75].

PFGE genotyping of the isolates revealed the existence of three PFGE clusters (A, B and C) (Figure 2). Each of them comprised enterotoxigenic isolates; cluster A included isolates from plant F only, whereas clusters B and C contained isolates from plant Z only. The assignment of isolates from the same processing plant into separate clusters indicates that *S. aureus* from different meat-processing establishments represent separate genetic populations. Furthermore, the findings of indistinguishable *S. aureus* pulsotypes from meat products, workers and equipment surfaces indicates probable cross-contamination pathways. These findings, in combination with the fact that isolates with no common *spa* type were detected from the two plants, supports the hypothesis that there is no universal spread of certain *spa* types and there is also a distinct genetic population of *S. aureus* in each processing plant. These facts emphasize the need for the strict application of good hygienic practices at the processing plant level in order to control the spread of foodborne and particularly multi-drug resistant pathogens to the community through the end products.

### 3.5. Next-Generation Sequencing

NGS was performed on two isolates, one MSSA belonging to the novel *spa* type, and one MRSA belonging to *spa* type t899. NGS analysis revealed that the isolate of the new *spa* type belonged to sequence type ST97 and the MRSA t899 isolate belonged to LA-associated ST398 (Table 3). The ST97 strain did not have any enterotoxin gene but carried the antibiotic efflux genes *mgr* (resistance to quinolones, tetracyclines), *arl*R (fluoroquinolones, disinfection agents and antiseptics), *mep*R (tetracycline, glycycycline), *nor*C (hydrophobic quinolones such as moxifloxacin and norfloxacin), *sdr*M (norfloxacin, ethidium bromide) and *sep*A (resistance to disinfectants and dyes). The ST398/t899 carried the same with ST97 antibiotic efflux genes plus the *nor*A (resistance to hydrophilic quinolones such as ciprofloxacin and norfloxacin) and the antimicrobial resistance genes *mec*A and *bla*Z (resistance to beta-lactamases), the *tet*M (tetracycline), the *fe*xA (phencicol antibiotics) and the *dfr*C (diaminopyrimidine antibiotic) [76]. In the international literature, it has been reported that the ST97 *S. aureus* clone is shared by a great number of different *spa* types. Zhang et al. [77] isolated 82 *S. aureus* ST97 from bovine clinical mastitis in China, which shared 15 different *spa* types (t359, t237, t4682, t521, t730, t16314, t16315, t224, t6297, t2756, t131, t1234, t267, UK-1). In a study contacted in Czech Republic, Pomorska et al. [78] reported that *S. aureus* isolates t267 and t359 from the blood of hospitalized patients belonged to the ST97 clone. Sato et al. [79] isolated five MRSA ST97 strains from pigs of one farm in Japan identified as *spa* type t1236. To our knowledge, this is the first time that the ST97 clone of *S. aureus* has been isolated in Greece.

## 4. Materials and Methods

### 4.1. Sample Collection

In the present study, we examined a total of 160 samples from two meat-processing establishments located in northern Greece (60 and 100 samples from establishment F and Z, respectively). The distance between the two establishments is more than 200 km. Thirty-seven samples were collected from the incoming meat (bovine, ovine, porcine), 24 samples from the final meat products (ground meat and non-ground meat products), 79 samples from the environment (22 from infrastructure surfaces that do not come in contact with meat and 57 from equipment surfaces) and 20 samples were obtained from the nasal cavities of workers (Table 1).

Surface sampling was performed by swabbing a minimum area of 100 cm^2^ (or the maximum available area, in case of smaller tools) using swabs moistened in buffered peptone water (LAB M, Lancashire, United Kingdom). A bi-lateral nasal (anterior nares) swab sample was taken from all workers (who participated voluntarily).

All samples were collected aseptically using sterile swabs along with single-use, screw-capped tubes filled with Stuart transport medium (Deltalab, Barcelona, Spain). Samples were transported to the laboratory under refrigerated conditions in less than four hours from the time of sampling.

### 4.2. Isolation and Identification of Staphylococcus aureus

Upon arrival to the laboratory, each sample was immediately transferred to a test tube filled with 10 mL of Tryptone Soy Broth (TSB, LAB M) supplemented with 6.5% (*w*/*v*) NaCl (Merck, Darmstadt, Germany) and 0.3% (*w*/*v*) yeast extract (LAB M). Following an 18 h incubation at 37 °C, 10 μL of the pre-enriched broth was surface-plated onto Baird–Parker Agar (LAB M) supplemented with egg-yolk tellurite (LAB M), and the plates were incubated aerobically at 37 °C for 48 h. Up to four presumptive *S. aureus* colonies (black colonies surrounded by an opaque zone and a clearance zone around the opaque zone) from each plate were sub-cultured on Tryptone Soya Agar (LAB M) for 24 h at 37 °C and were then subjected to Gram staining, along with mannitol fermentation testing and catalase-testing [80]. Furthermore, all suspect colonies were subjected to a rapid test (Microgen Staph Rapid Test; Microgen Bioproducts, Surrey, UK) for the detection of the coagulase enzyme and of the protein A, assisting the tentative identification to the species level (*S. aureus*). Among them, one presumptive *S. aureus* isolate (Gram-positive, catalase-positive, mannitol-positive, coagulase-positive and protein A-positive) per sample was randomly chosen and stored under freezing conditions (−80 °C) in cryotubes containing TSB with 20% glycerol for further processing.

### 4.3. Molecular Characterization of Staphylococcus aureus

For species confirmation of the presumptive *S. aureus* isolates, a PCR test detecting the *coa* and the species-specific *nuc* genes was performed. Genomic DNA was extracted by using a DNA purification protocol for Gram-positive bacteria (Pure Link Genomic DNA kit; Invitrogen, Carlsbad, CA, USA), while PCR conditions [81] and primers [82,83] previously described were used (Supplementary Table S1).

### 4.4. Detection of Virulence Genes and Methicillin Resistance Genes

Multiplex PCRs were used for the detection of virulence genes contributing to the pathogenicity of *S. aureus* and for the detection of methicillin resistance genes. Two separate sets of primers and multiplex PCR reactions were used to assess the carriage of genes that encode nine types of SEs (sea, seb, sec, seh and sej; sed, see, seg, sei) and the carriage of the tst gene, which encodes for the production of the toxic shock syndrome toxin-1 (TSST-1) [84]). The carriage of the *mec*A and *mec*C genes, which confer resistance to methicillin and the carriage of the *luk*F-PV gene, which encodes for the Panton–Valentine Leukocidin toxin (PVL) were sought also via multiplex PCR using the primers and conditions described by Stegger et al. [85] (Supplementary Table S1).

### 4.5. Antimicrobial Susceptibility Testing

Antimicrobial susceptibility testing was performed by the disc-diffusion method using Mueller–Hinton agar (Merck) according to Clinical and Laboratory Standard Institute Performance Standards for Antimicrobial Susceptibility Testing [86]. For each isolate, the inocula were prepared by adjusting the turbidity to 0.5 McFarland. Susceptibility was evaluated to the following 14 antimicrobials: the beta-lactam antibiotics penicillin 10 IU (P), ampicillin 10 μg (AMP), cefoxitin 30 μg (FOX) and amoxicillin/clavulanic acid 20/10 μg (A/C); tetracycline 30 μg (TET); the aminoglycosides gentamicin 10 μg (GEN), tobramycin 10 μg (ΤOΒ) and amikacin 30 μg (AMK); the macrolide erythromycin (E) 10 μg; the lincosamide clindamycin 2 μg (DA); ciprofloxacin 5 μg (CIP) belonging to quinolones; chloramphenicol 30 μg (C) belonging to amphenicol class; fusidic acid 10 μg (FU) belonging to fusidanes; trimethoprim/sulfamethoxazole 1.25/23.75 μg (SXT) belonging to diaminopyrimidine/sulfonamide classes. *S. aureus* ATTC 25923; *Escherichia coli* ATCC 25922 and *Enterococcus faecalis* ATCC29212 were used as control strains. Multidrug resistance (MDR) was defined as acquired non-susceptibility to at least one agent in three or more antimicrobial categories [87]. *S. aureus* ATTC 25923; *Escherichia coli* ATCC 25922 and *Enterococcus faecalis* ATCC29212 were used as control strains.

### 4.6. Assessment of Biofilm-Formation Ability

The ability of the *S. aureus* isolates to produce biofilms was evaluated following the protocol of the microtiter plate method, as described by Wang et al. [88]. Staphylococcal strains were characterized according to Borges et al. [89] as no biofilm producers, weak biofilm producers, moderate biofilm producers, or strong biofilm producers.

### 4.7. Pulsed Field Gel Electrophoresis Analysis

PFGE analysis of the *S. aureus* isolates was performed according to a standardized protocol [90] with the *Sma*I endonuclease (New England Biolabs, Beverly, MA, USA) using a CHEF-DR III apparatus (Bio-Rad Laboratories Inc., Hercules, CA, USA). DNA from *Salmonella enterica* serotype Braenderup H9812, which was digested with *Xba*I, was used as a reference size standard, while PFGE patterns were analyzed using the FPQuest software (Bio-Rad Laboratories Inc. Pty Ltd.). PFGE profiles were compared using the Dice correlation coefficient with a maximum position tolerance of 1.5% and an optimization of 1.5%, while the Unweighted Pair Group Method using Averages (UPGMA) was used for clustering analysis and the generation of a dendrogram.

### 4.8. Spa Typing

For amplification of the Staphylococcus protein A (spa) repeat region, the extracted DNA (from Section 4.3) was subjected to a PCR using the primers described by Aires-de-Sousa et al. [91] according to the protocol described in the Ridom Spa Server website (http://spaserver.ridom.de/ accessed on 2 March 2021). PCR products were sequenced in a 3130 genetic analyzer (Thermo Fisher Scientific, South San Francisco, CA, USA). For the analysis of *spa* sequences, the software Ridom StaphTypeTM (Ridom GmbH, Wόrzburg, Germany) was used [92].

### 4.9. Next-Generation Sequencing and Analysis of the Whole Genome

Two isolates were selected for further analysis by next-generation sequencing: *S. aureus* isolate was assigned to the novel spa type (Z77) and the multi-resistant MRSA isolate (Z112). DNA was extracted using the DNA extraction kit (Qiagen, Hilden, Germany), and its concentration was measured using the Qubit double strand (ds) DNA HS assay kit (Q32851, Life Technologies Corporation, Grand Island, NY, USA). NGS was performed using the Ion PGM Hi-Q View Sequencing kit on an Ion Torrent PGM Platform (Life Technologies Corporation).

The procedures of shearing, purification, ligation, barcoding, size selection, library amplification and quantitation, emulsion PCR and enrichment were performed according to the manufacturer’s instructions. PCR products were loaded on an Ion-316™ chip kit V2BC. The Ion PGM Hi-Q View (200) chemistry (Ion PGM Hi-Q View Sequencing kit) was applied. The sequence reads were de novo assembled and annotated using Geneious Prime version 2021.2.1. Sequences from the whole genome of the samples Z77 and Z112 were submitted to ENA and received the accession numbers ERS13643805 and ERS13643806, respectively.

MLST analysis was performed using the free web-based *S. aureus* database in PubMLST (https: //pubml st.org/saure us/ accessed on 10 March 2021) [93]. Antimicrobial resistance genes were identified using the online databases Resfinder version 4.1 [94] and Comprehensive Antibiotic Resistance Database (CARD) [76], with the selection criteria set to perfect (100% identity) and strict (>95% identity) hits only to the reference sequences in CARD [95]. Virulence genes and plasmid content were identified using the VirulenceFinder 2.0 and PlasmidFinder version 2.1 from the Center for Genomic Epidemiology (http://www.genomicepidemiology.org/ accessed on 15 March 2021) [96]. The threshold for %ID and for the minimum length were set to 90% and 60%, respectively.

## 5. Conclusions

Despite the limitations of the present study (two meat processing establishments and a relatively low number of examined samples), interesting conclusions can be drawn from the results. This study revealed the low prevalence of MRSA and MDR *S. aureus*, no common palsotypes among the isolates from the two establishments and high genetic diversity by spa typing. Although the prevalence of MRSA was low, meat-processing establishments are a very important spot for controlling pathogen spread in the community. Thus, the implementation of rules of good hygiene practice in combination with biosecurity measures are crucial factors for achieving this goal. However, further data are needed to monitor the spread of MRSA at meet the meat processing establishments level.

## Figures and Tables

**Table 1:** Isolation frequency of *Staphylococcus aureus* and of methicillin-resistant *S. aureus* (MRSA) from the incoming meat, raw meat products, the environment, and the workers of two meat-processing plants in northern Greece.

Sample Origin	Plant F			Plant Z			Total		
	*n* *	*S. aureus* ± (%)	MRSA (%)	*n* *	*S. aureus* ± (%)	MRSA (%)	*n* *	*S. aureus ±* (%)	MRSA (%)
Incoming bovine meat	6	1 (16.7)	-	6	-	-	12	1 (8.3)	-
Incoming porcine meat	6	1 (16.7)	-	14	1 (7.1)	-	20	2 (10.0)	-
Incoming ovine meat	4	-	-	1	-	-	5	-	-
Non-ground meat products	6	-	-	2	-	-	8	-	-
Ground meat products	8	4 (50.0)	-	8	2 (25.0)	1 (12.5)	16	6 (37.5)	1 (6.3)
Nasal cavity of workers	9	3 (33.3)	-	11	2 (18.2)	-	20	5 (25.0)	-
Infrastructure	5	-	-	17	1 (5.9)	-	22	1 (4.5)	-
Equipment	16	5 (31.3)	-	41	2 (4.9)	-	57	7 (12.3)	-
Total	60	14 (23.3)	-	100	8 (8.0)	1 (1.0)	160	22 (13.8)	1 (0.6)

*n* *, number of samples; ±, includes MRSA isolates.

**Table 2:** Characteristics of *Staphylococcus aureus* and of methicillin-resistant *S. aureus* (MRSA) strains isolated from different sites within two meat-processing establishments in northern Greece.

Sample Code *	Sample Origin	Antimicrobial Resistance Profile **	*mec*A	*mec*C	*luk*F-PV*/tst*	SE Genes	Biofilm Formation Ability	*Spa* Type	PFGE Electrophoretic Cluster
F20	Meat	P, TET, TOB	-	-	-/-	-	moderate	t084	A
F22	Equipment	-	-	-	-/+	*sed, sei*	moderate	t012	NT ***
F28	Meat product	P, A/C, TET	-	-	-/-	-	moderate	t499	A
F29	Meat product	P, TET	-	-	-/-	-	moderate	t774	A
F32	Meat product	P	-	-	-/-	*seb*	moderate	t084	A
F36	Equipment	P, A/C	-	-	-/-	*seb*	moderate	t084	A
F37	Equipment	P	-	-	-/-	*seb*	moderate	t084	A
F39	Equipment	P, A/C	-	-	-/-	*seb*	moderate	t084	A
F40	Equipment	P, AMP, A/C, TOB	-	-	-/-	*seb*	moderate	t084	A
F42	Meat product	P, A/C	-	-	-/-	*seb*	moderate	t084	A
F43	Meat product	P, A/C	-	-	-/-	*seb*	moderate	t084	A
F44	Human nasal cavity	P	-	-	-/-	*sed, sei*	moderate	t891	NT
F45	Human nasal cavity	P	-	-	-/-	*sed*	moderate	t197	A
F46	Human nasal cavity	P	-	-	-/-	*seb*	moderate	t084	NT
Ζ5	Human nasal cavity	P, A/C	-	-	-/+	*seb, sed, sei*	moderate	t1510	NT
Ζ7	Human nasal cavity	-	-	-	-/-	-	moderate	new	C
Ζ18	Infrastructure	-	-	-	-/-	-	moderate	new	C
Ζ19	Equipment	-	-	-	-/-	-	moderate	t091	B
Ζ77	Meat product	-	-	-	-/-	-	moderate	new	C
Ζ104	Equipment	-	-	-	-/-	-	strong	t091	B
Ζ112	Meat product	P, AMP, A/C, FOX, TET, TOB, C, SXT	+	-	-/-	-	moderate	t899	NT ***
Ζ146	Meat product	-	-	-	-/-	-	moderate	new	C

* F: Plant F, Ζ: Plant Ζ. ** (P). penicillin, (AMP) ampicillin, (A/C) amoxicillin/clavulanic acid, (TET) tetracycline, (FOX) cefoxitin, (GEN) gentamicin, (AMK) amikacin, (ΤOΒ) tobramycin, (E) erythromycin, (DA) clindamycin, (CIP) ciprofloxacin, (C) chloramphenicol, (FU) fucidic acid, (SXT) sulfamethoxazole/trimethoprim. *** NT: Isolates non-typeable by PFGE.

**Table 3:** Results of the analysis of the whole genome sequences of the two *S. aureus* isolates subjected to NGS.

Feature	Isolate Z77	Isolate Z112
Number of contigs	85	309
N50 (bp)	169,989	134,669
GC (%),	33.0	33.1
Length (bp)	2,896,939	2,840,021
Sequence type (ST)	97	398
Spa type	Novel type (repeats 07-23-12-12-21-17-13-34-33-34)	t899
Resistance genes	-	*mec*A, *bla*Z, *tet*M, *fex*A, *dfr*C
Antibiotic efflux genes	*mgr*A, *arl*R, *mep*R, *nor*C, *sdr*M, *sep*A	*mgr*A, *arl*R, *mep*R, *nor*A, *nor*C, *sdr*M, *sep*A
Toxin genes	*hlg*A, *hlg*B, *hlg*C, *luk*D, *luk*E	*hlg*A, *hlg*B, *hlg*C
Exoenzyme genes	*aur*, *spl*A, *spl*B, *spl*E	*aur*
Host immune defense genes	*sak*, *scn*	-
Plasmids	rep20	rep21, repUS43

## Data Availability

Not applicable.

## Supplementary Materials

The following supporting information can be downloaded at: https://www.mdpi.com/article/10.3390/pathogens11111370/s1, Table S1: Primers used in this study for the detection of molecular characterization genes, toxin genes and methicillin resistance genes.
