# Supplementary material for: Prevalence, Infectious Characteristics and Genetic Diversity of Staphylococcus aureus and Methicillin-Resistant Staphylococcus aureus (MRSA) in Two Raw-Meat Processing Establishments in Northern Greece
Source: Pathogens. 2022 Nov 17;11(11):1370. doi: 10.3390/pathogens11111370 (PMC9697755; doi:10.3390/pathogens11111370)
Supplement: Supplementary file 1 [file pathogens-11-01370-s001.zip › pathogens-1942993-supplementary.pdf]

Table S1. Primers used in this study for the detection of molecular characterization genes, toxin genes and methicillin resistance genes

	Primers*	Primer sequence (5′-3′)	Amplified product size (bp)	Reference
**Molecular characterization genes**				
	coa F	ATAGAGATGCTGGTAAGG	500-650	[82]
	coa R	GCTTCCGATTGTTCGATGC		
	nuc F	GGCAATTGTTTCAATATTAC	416	[83]
	nuc R	TTTTATTTGCATTTTCTACC		
**Toxin genes**				
	sea F	GCAGGGAACAGCTTTAGGC	521	[84]
	sea R	GTTCTGTAGAAGTATGAAACACG		
	seb F	ACATGTAAT TTT GATATTCGCACTG	667	[84]
	seb R	TGCAGGCATCATGTCATACCA		
	sec F	CTTGTATGTATGGAGGAATAACAA	284	[84]
	sec R	TGCAGGCATCATATCATACCA		
	sed F	GTGGTGAAATAGATAGGACTGC	385	[84]
	sed R	ATATGAAGGTGCTCTGTGG		
	see F	TACCAATTAACTTGTGGATAGAC	171	[84]
	see R	CTCTTTGCACCTTACCGC		
	seg F	CGT CTC CAC CTG TTG AAG G	328	[84]
	seg R	CCA AGT GAT TGT CTA TTG TCG		
	seh F	CAA CTG CTG ATT TAG CTC AG	359	[84]
	seh R	GTC GAA TGA GTA ATC TCT AGG		
	sei F	CAA CTC GAA TTT TCA ACA GGT ACC	466	[84]
	sei R	CAG GCA GTC CAT CTC CTG		
	sej F	CAT CAG AAC TGT TGT TCC GCT AG	142	[84]
	sej R	CTG AAT TTT ACC ATC AAA GGT A		
	tsst F	GCT TGC GAC AAC TGC TAC AG	559	[84]
	tsst R	TGG ATC CGT CAT TCA TTG TTA T		
	pvl F	GCTGGACAAAACTTCTTGGAATAT	83	[85]
	pvl R	GATAGGACACCAATAAATTCTGGATTG		

**Methicillin resistance genes**				
	mecA F	TCACCAGGTTCAAC[Y]CAAAA	162	[85]
	mecA R	CCTGAATC[W]GCTAATAATATTTC		
	mecC F	GAAAAAAAGGCTTAGAACGCCTC	718	[85]
	mecC R	CCTGAATC[W]GCTAATAATATTTC		

* F: forward, R: reverse
